# Chicago Medical Society

**Published:** 1868-06

**Authors:** 


### CHICAGO MEDICAL SOCIETY.

Friday Evening, April 17, 1868.

The Society was called to order by the President, and the minutes of the previous meeting read and approved.

Dr. Loverin reported favorably on the application of Dr. T. S. Bidwell, when he was balloted for and duly elected a member of the Society.

Dr. Bogue presented his report on the sanitary condition of the North Division of the city, for three months, commencing January 1, 1868.

On motion of Dr. Loverin, the report of Dr. Bogue was accepted.

Dr. Mitchell exhibited to the Society a fibrous polypus of the uterus. After removal of the tumor the hemorrhage was but slight, the loss of blood being only two drachms. The patient became twice pregnant while the tumor was in the cavity of the uterus.

Dr. Bogue related the case of an Italian who had been stabbed, from which he removed some two and a-half inches of the blade of a heavy dirk, found imbedded between the fourth and fifth dorsal vertebræ. Supposed the injury did not involve the cord, but probably the meninges. Prior to removal, commenced having paralysis. The bladder could not be easily evacuated without pressure over the part; constipation, but not obstinate; had some cough. Patient now able to move about the ward by the use of two canes, and is doing well.

Dr. Lyman reported the results of his examination upon the pathological specimen presented by Dr. Wanzer, at a previous meeting. Found it to be composed of yellow fibrous tissue. Also reported a case of nephritic colic, relieved by the administration of chloroform; found in the urine a calculus, which was exhibited to the Society.

On motion of Dr. Wanzer, the report of Dr. Lyman was accepted and ordered to be placed on file.

Dr. Davis read his report on the sanitary condition of the city, which, on motion of Dr. Loverin, was accepted.

Dr. Miller remarked, he fully accorded with Dr. Davis' report, and expressed his desire to have such portions of the report as related to the sanitary condition published in the daily papers of the city.

Dr. Hatch said that he had seen a number of cases of vaccination, where severe inflammation was the result; was confident it was not owing to the vaccine used, as he was satisfied it was pure and reliable; also accorded with Dr. Davis in his report, and hoped it would be brought before the public.

Dr. Lyman stated that he had also seen the same results as expressed by Dr. Hatch, in a number of instances where the same vaccine had been used, some of which progressed favorably, while others had a severe type of inflammation.

The President reported the result of the vaccination of a lady and her two children, where the same vaccine had been used; the children terminating well, while the mother, unaccountably, the opposite.

Dr. Davis was of the opinion that, during the past winter, there had been a predisposing tendency to an unhealthy inflammation; thinks it obvious we will have such seasons, and therefore recommends parents to have their children vaccinated during healthy ones.

Dr. Ross said that, in his section of the city, he had seen a larger number of cases of rubeola than usual. Had noticed many cases of vaccination having an inflammatory tendency.

Dr. Mitchell made the following motion, *viz.:* That Dr. Davis be requested to prepare for publication in the public journals, so much of his sanitary report as relates to drainage, and other matters which he deems essential to bring before the public.

Dr. Lyman said, if the report was to be published, it should contain nothing that would cast reflections on the Health Department.

Dr. Trimble fully accorded with Dr. Lyman. Had made a complete survey of his district, and found it in as good condition as could be expected.

Dr. Paoli considered the Board of Health ought to be thankful for the information given, as contained in the report of Dr. Davis, being aware of many streets being in a very filthy condition, more especially Bremer Street.

Dr. Davis said there was no member of the Society that would find less fault with the Board of Health than himself, but he did complain that they had not a more perfect system.

On the motion of Dr. Mitchell being submitted to the Society, it was unanimously carried.

The Society then adjourned.

---

Friday Evening, April 24, 1868.

The regular meeting of the Chicago Medical Society was called to order by the President. The Secretary then read the proceedings of the last meeting, which were approved and placed on file.

Dr. Wanzer presented a pathological specimen, in form of a fœtus about seven months old. The patient had been flooding for two months previous to delivery, losing each day about the same as during her menstrual period. The true labor pains commenced eight days before delivery. The nates presented, and the fœtus was delivered without rupture of the membranes, the cord being around the neck. The constant discharge which the patient had suffered was ascribed to an attack of measles, leaving her with a severe cough. When called, there was considerable hemorrhage, which ceased soon after delivery. The patient is a strong, robust woman. Had she not have been, the case would probably have resulted unfavorably in this rare case of placenta prævia. Under the use of tinc. ferri chloridi, she is rapidly improving.

Dr. Loverin remarked that it was undoubtedly a case of placenta prævia. Stated that he had a case some years ago; when called, there was severe hemorrhage, which nearly proved fatal.

Dr. Paoli remarked that, in placenta prævia, the object is to deliver before there is hemorrhage, as in nearly all cases the os uteri is well dilated.

Dr. Wickersham cited a case, in the West Division, where hemorrhage proved nearly fatal, while the os uteri was rigid.

Dr. Hildreth reported a case of catarrhal ophthalmia, occurring at the County Hospital. The globe of the eye continued to enlarge, until it was two-thirds the size of a hen's egg and resembled malignant disease. Found slight syphilitic eruption on one hand. The sight of the eye being previously destroyed, an incision was made in the eye, but little serum and blood escaping. The patient was placed upon the following treatment;

℞. Hydrarg. Bichlor.,---------------------- gr. j

Potassæ Iodidi,------------------------ ʒiv

Aqua, -------------------------------- ℥viij

M. S., teaspoonful every four hours.

In ten days, the eye had contracted to a small stump, the cornea, lens, and humor having sloughed before the enlargement occurred. Thinks that had the anti-syphilitic remedies been employed early, the eye might have been saved.

Dr. Andrews exhibited an endoscope to the Society, for the diagnosis and treatment of chronic gonorrhœa, which instrument was examined by all present.

Dr. Paoli returned thanks to Dr. Andrews for the privilege of examining the instrument.

Dr. Wickersham mentioned a case of gonorrhœa, occurring from eating grapes, and always returning when that fruit was eaten.

Dr. Wanzer made some inquiries regarding the use of forceps in obstetrical practice, which was participated in by Drs. Loverin and Paoli.

Dr. G. Fredericke was then proposed for membership. The Society then adjourned.

---

May, 1, 1868.

The regular meeting of the Chicago Medical Society was called to order by the President, Dr. Marguerat. The Secretary read the proceedings of the last meeting, which were duly approved.

Dr. Loverin recommended the name of Dr. G. Fredericke for membership, and on order of the President, the Society proceeded to ballot, and Dr. Fredericke was duly elected a member of the Society.

The President also recommended Dr. Brummind, House-Surgeon at Mercy Hospital, for membership.

Dr. Loverin opened the discussion of conjunctivitis, which was the order for the evening, by giving all the forms and the treatment for the different stages of the disease; and, although interesting and profitable, it elicited no facts of importance not already before the profession. The discussion was participated in by Drs. Holmes, Clarke, Paoli, and Marguerat.

Dr. Holmes remarked that conjunctivitis was one of the worst diseases we have to contend with, some apparently mild cases terminating unfavorably, therefore rendering the prognosis difficult. Remarked that the use of ice-water, *constantly* applied to the eyes, was of much importance, recommending it to be used under observation of a good nurse. Recommends in the acute stage of conjunctivitis, the application of strong solutions of nitrate of silver, say from half a drachm to two scruples to the ounce of water in adults, and from eight to ten grains in infants. Seldom resorts to bleeding, but gives veratrum viride internally, as well as in the form of a lotion. Unless the eye is very vascular and has a dark red appearance, does not think it best to scarify.

Dr. Clarke remarked, that he had more confidence in the abstraction of blood in conjunctivitis than any other remedy. Cited the case of a nephew of his, having applied six leeches, followed by severe hemorrhage, suffering for some ten days from loss of blood. After the bleeding, however, the eyes were perfectly well.

Dr. Paoli remarked that he did not approve of the administration of tartar emetic (tartarized antimony), as recommended by Dr. Loverin, nor bleeding, but recommended diaphoretics, foot-baths, etc. He also protested against the use of strong solutions of nitrate of silver in the first stage of conjunctivitis.

Dr. Marguerat remarked, that he had observed very beneficial results in some of Dr. Holmes practice from the use of strong solutions of nitrate of silver.

Dr. Holmes remarked, that the patients afflicted with conjunctivitis usually came to him at a late day, but if they would come early he would give them a warm bath and veratrum viride for the first twenty-four hours. Also remarked that the majority of cases of chronic conjunctivitis become anæmic, requiring tonics with other remedies.

The Society then passed to miscellaneous business.

The subject chosen for next discussion was: "Disproportion of the Fœtus to the Pelvis, and the Mode of Treatment." Leaders: Drs. Reid and Paoli.

Society adjourned.

